# Pro- and Anti-Inflammatory Cytokine Balance in Major Depression: Effect of Sertraline Therapy

**DOI:** 10.1155/2007/76396

**Published:** 2008-01-20

**Authors:** Levent Sutcigil, Cagatay Oktenli, Ugur Musabak, Ali Bozkurt, Adnan Cansever, Ozcan Uzun, S. Yavuz Sanisoglu, Zeki Yesilova, Nahit Ozmenler, Aytekin Ozsahin, Ali Sengul

**Affiliations:** ^1^Department of Psychiatry, Gülhane Military Medical Academy, 06018 Ankara, Turkey; ^2^Division of Internal Medicine, GATA Haydarpasa Training Hospital, 34668 Istanbul, Turkey; ^3^Department of Immunology, Gülhane Military Medical Academy, 06018 Ankara, Turkey; ^4^Department of Monitoring and Evaluation, Turkish Ministry of Health, 06570 Ankara, Turkey; ^5^Department of Internal Medicine, Gülhane Military Medical Academy, 06018 Ankara, Turkey

## Abstract

The specific associations between antidepressant treatment and alterations in the levels of cytokines remain to be elucidated. In this study, we aimed to explore the role of IL-2, IL-4, IL-12, TNF-α, TGF-β1, and MCP-1 in major depression and to investigate the effects of sertraline therapy. Cytokine and chemokine levels were measured at the time of admission and 8 weeks after sertraline treatment. Our results suggest that the proinflammatory cytokines (IL-2, IL-12, and TNF-α) and MCP-1 were significantly higher, whereas anti-inflammatory cytokines IL-4 and TGF-β1 were significantly lower in patients with major depression than those of healthy controls. It seems likely that the sertraline therapy might have exerted immunomodulatory effects through a decrease in the proinflammatory cytokine IL-12 and an increase in the anti-inflammatory cytokines IL-4 and TGF-β1. In conclusion, our results indicate that Th1-, Th2-, and Th3-type cytokines are altered in the depressed patients and some of them might have been corrected by sertraline treatment.

## 1. INTRODUCTION

There is now evidence that major depression is accompanied by significant changes in cell-mediated and humoral immunity, and these changes may be related to the pathophysiology or pathogenesis of that illness (Connor and Leonard [1], Dantzer et al. [2], Kim et al. [3], Licinio and Wong [4]), yet data are inconsistent. Some studies have shown that major depression is associated with dysregulation of immune mediators, such as the rise in interleukin (IL)-1*β*, IL-6, IL-12, soluble IL-6R, IL-2, soluble IL-2R, IL-1Ra, and IFN-*γ* (Kaestner et al. [5], Kim et al. [3], Maes et al. [6], Maes et al. [7], Maes et al. [8], Maes [9], Maes et al. [10], Seidel et al. [11]). However, conflicting results have also been described (Brambilla and Maggioni [12], Brambilla et al. [13], Carpenter et al. [14], Rothermundt et al. [15]). These changes have been considered in terms of the imbalance between individual pro- and anti-inflammatory cytokines and the T helper 1 (Th1) and T helper 1 (Th2) imbalance in major depression. On the other hand, an enhanced secretion of such proinflammatory cytokines would not only lead to activation of T and B lymphocytes, but also could affect the brain and elicit various symptoms of depression, such as loss of appetite, listlessness, and sleep disturbances (Maes [16]). Furthermore, only few clinical studies report whether the patients that are included in the sample receive antidepressant treatment or not. Most of these studies are inconsistent and based on data generated from ex vivo or in vitro immunological evaluations showing that the antidepressant treatment usually normalizes the changes in both cellular and humoral immunity that occur in depression (Neveu and Castanon [17]). Although the precise mechanism of how the antidepressants act is uncertain, there is some other evidence in the literature suggesting that they could reduce the release of pro-inflammatory cytokines and other immunological factors (Lanquillon et al. [18], Leonard [19]). It has been reported that antidepressants can decrease the Th1/Th2 or proinflammatory/anti-inflammatory cytokine ratio (Kubera et al. [20], Maes [21]) and it has been hypothesized that cytokine hypersecretion may be involved in the pathophysiology of depressive disorders (Leonard [19]). Nevertheless, treatment of depressive disorders with antidepressants is not always accompanied by a reduction in the concentrations of proinflammatory cytokines (Weizman et al. [22], Maes [16]). Thus, the specific associations between antidepressant treatment and alterations in the levels of cytokines remain to be elucidated.

Classically, IL-2 and IL-12 are Th1 type proinflammatory cytokines, but IL-4 and transforming growth factor beta (TGF- *β*) are Th2 and Th3 type anti-inflammatory cytokines, respectively. These type cytokines are secreted by T lymphocytes while tumor necrosis factor-alpha (TNF-*α*) generally secreted by monocytes and macrophages which is the other pro-inflammatory cytokine. The goals of this study are (1) to determine the IL-2, IL-4, IL-12, TNF-*α*, TGF beta-1 (TGF-*β*1), and monocyte chemotactic protein-1 (MCP-1) in our sample of depressed patients, (2) to evaluate whether these characteristics differ between patients with major depression and age- and sex-matched healthy controls, and (3) to investigate the effect of treatment with, a selective serotonin reuptake inhibitor (SSRI) antidepressant, sertraline on these parameters.

## 2. MATERIALS AND METHODS

Our study included 30 only first episode unipolar depressive outpatients consecutively admitted to our psychiatry department. Two patients dropped out of the study because acute medical disease. Five patients who fail to respond to sertraline therapy were also excluded at the end of the study. Twenty three (11 females, 12 males) patients have completed the study. Twenty five healthy volunteers (12 females, 13 males) were included the study as controls. Informed consent was obtained from all subjects. The study protocol was approved by the ethic committee of Gülhane Military Medical Academy.

All depressed patients fulfilled the DSM-IV criteria for major depressive episode. The patients have been seen by a psychiatrist in the outpatient unit of our department. Those who were meeting the criteria of the research have been referred to the primary investigator (PI) who is also a psychiatrist. The healthy controls have been recruited from the employees of the medical academy. Initially, these subjects are selected after detailed physical and mental examinations. Controls which met the inclusion criteria were interviewed by the PI similar as the patients. Controls were matched to the subjects according to their age and gender.

The severity of the depression was quantified by the Hamilton Depression Rating Scale (HDRS) on pre- and posttreatment with sertraline (50–100 mg/day, 8 weeks). The pre- and post-HDRS assessments were conducted only by the PI. Clinical and laboratory data were anonymous. All ratings were evaluated by the PI blinded to the immunological profiles. All parameters were repeated after the treatment with sertraline. After treatment, the patients which were no longer meeting diagnostic criteria of unipolar major depressive disorder have been accepted as responders. The response rate was 77%.

Exclusion criteria were any additional axis I or axis II DSM-IV diagnosis, current pregnancy, acute or chronic infections within the past month, autoimmune, allergic, neoplastic, or endocrine diseases and other acute physical disorders, including surgery or infarction of the heart or brain within the past 3 months. Patients exposed to any drug including antidepressants, nonsteroidal anti-inflammatory drugs, and oral contraceptives in the past 6 weeks were also excluded. Healthy volunteers were also interviewed and in addition to the above exclusion criteria, those with no lifetime or current diagnosis of any psychiatric disorders were included as the control group.

After an overnight fast, blood samples for the assays were collected at 9 a.m. (±30 minutes) from patients with major depression and healthy volunteers. Ten millilitres venous blood was drawn and centrifuged at 3000 rpm for 10 minutes for the measurements of IL-2, IL-4, IL-12, TGF-*β*1, TNF-*α*, and MCP-1 levels. All sera samples were stored at –70°C until run. All immunological parameters were determined by Enzyme Immunoassay method with Bender MedSystems kits (Bender MedSystems GmbH Campus Vienna Biocenter 2 A-1030, Vienna, Austria). According to kit prescription, the intra- and interassay coefficients of variation (CVs) were 4.7% and 8.7% for MCP-1, 6.7% and 8.5% for TGF-*β*1, 3.0% and 4.8% for IL-12, 5.2% and 8.0% for IL-2, 4.8% and 5.6% for IL-4, and 6.9% and 7.4% for TNF-*α*, respectively.

The same procedures have been applied during post-treatment assessment. The duration of treatment was minimum 8 weeks and posttreatment assessment has been performed in the first week after end of the 8th week.

## 3. STATISTICAL ANALYSIS

Data were analyzed with SPSS (SPSS Inc., Ill, USA) statistical software. Descriptives were quoted as the mean ± SD. Changes in HDRS and immune measurements were calculated. The relations among these changes were assessed with Spearman's rho coefficient of correlation. Before and after values of the parameters were compared with “paired samples *t* test.” We used “independent samples *t* test” to compare the parameter values of the control and treatment groups. For multiple tests, we used Bonferroni correction. Pearson coefficient of correlation for the parameters was calculated. A *P* value less than or equal to .05 was evaluated as statistically significant.

## 4. RESULTS

The clinical and immunological characteristics of the patients with major depression and healthy controls and the results of comparisons are shown in Table 1. There was no difference between both patient groups and controls with respect to age (*P* = .835) and sex (*P* = .945). The mean levels of HDRS (*P* < .001), IL-2 (*P* < .001), IL-12 (*P* < .001), TNF-*α* (*P* < .001), and MCP-1 (*P* < .001) were significantly higher in patients with major depression than in controls. As compared with the controls, the mean levels of IL-4 (*P* < .001) and TGF-*β*1 (*P* < .001) were significantly lower in patient group. The nonresponders to sertraline have not been included to the study, since the number of nonresponders was only five which could not be included in statistical analysis.

**Figure 1:**
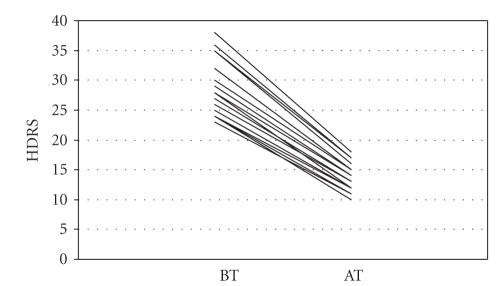
Hamilton depression rating scale (HDRS) levels before (BT) and after (AT) sertraline treatment.

**Figure 2:**
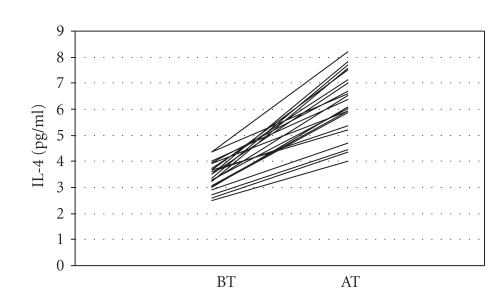
Interleukin-4 (IL-4) levels before (BT) and after (AT) sertraline treatment.

**Figure 3:**
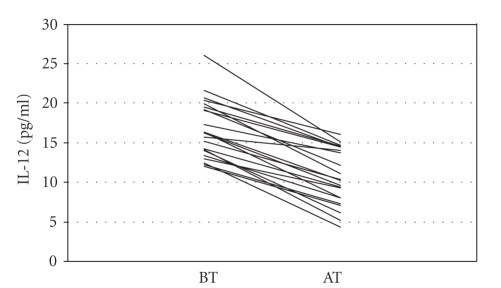
Interleukin-12 (IL-12) levels before (BT) and after (AT) sertraline treatment.

**Figure 4:**
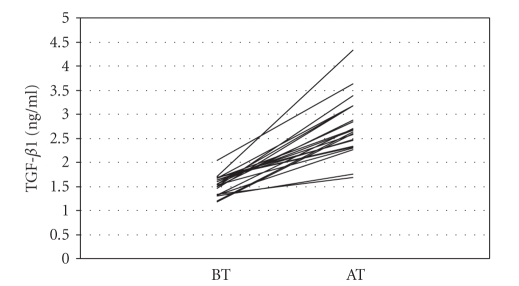
Transforming growth factor *β*1 (TGF- *β*1) levels before (BT) and after (AT) sertraline treatment.

In the patient group, clinical and immunological characteristics before and after treatment with sertraline and the results of comparisons are given in Table 2. The mean levels of HDRS (*P* < .001), TNF-*α* (*P* < .001), MCP-1 (*P* < .001), IL-2 (*P* < .001), and IL-12 (*P* < .001) decreased significantly after the sertraline treatment, whereas IL-4 (*P* = .001) and TGF-*β*1 (*P* < .001) increased significantly. However, only reduction of HDRS (*r* = 0.966; *P* < .001, Figure 1) and IL-12 (*r* = 0.837; *P* < .001, Figure 3) and increment of IL-4 (*r* = 0.631; *P* = .001, Figure 2) and TGF-*β*1 (*r* = 0.524; *P* = .010, Figure 4) after therapy were found to be correlated with sertraline treatment. The reduction of IL-2, TNF-*α*, and MCP-1 after treatment did not correlate significantly with sertraline therapy.

Change in HDRS and the other immune measurements were not significantly correlated. There was a positive correlation between both changes in IL-12 and IL-2 (*ρ* = 0.792; *P* < .001).

## 5. DISCUSSION

In this study, we aimed to explore the role of IL-2, IL-4, IL-12, TNF-*α*, and TGF-*β*1, which represent the cytokines of the Th1, Th2, and Th3 types, and MCP-1 in major depression, and to investigate the effects of sertraline therapy. Our results suggest that the proinflammatory cytokines (IL-2, IL-12, and TNF-*α*) and MCP-1 were significantly higher, whereas anti-inflammatory cytokines IL-4 and TGF-*β*1 were significantly lower in patients with major depression than those of healthy controls. It seems likely that the sertraline therapy might have exerted immunomodulatory effects through a decrease in the proinflammatory cytokine IL-12 and an increase in the anti-inflammatory cytokines IL-4 and TGF-*β*1.

In agreement with our findings, previous studies in major depressive disorder report that the elevation in IL-2 production during depressive state (Seidel et al. [23], Schlatter et al. [24]). This IL-2 overproduction could be integrated in the inflammatory response system, which is activated during depression, and is consistent with the shift Th1/Th2 mechanism. In addition, in vitro studies with human whole blood have reported that sertraline is able to inhibit the production of IL-2 (Kubera et al. [25], Kubera et al. [20]). Conversely, other authors reported decreased production IL-2 in major depression (Pavón et al. [26]) and a normalizing trend after treatment (Kanba et al. [27], Weizman et al. [22]). The reason for the discrepancy between these studies is unclear, and due to the numerous methodological dissimilarities between studies, it is difficult to speculate.

As others (Myint et al. [28]), we demonstrated that depressed patients had lower IL-4 levels compared to normal controls. Nevertheless, Pavón et al. [26] reported higher IL-4 levels in patients with major depression than controls, while others observed unchanged IL-4 levels (Natelson et al. [29], Schlatter et al. [24]). On the other hand, in contrast to our findings, Myint et al. [28] reported that IL-4 levels were reduced in depressed patients after the 8-week treatment. Differences among studies are likely to be attributable to factors such as patient diagnosis, diagnostic criteria utilized, patient demographic, and the immune measured assayed.

Another finding of our study, like others (Kim et al. [3], Lee and Kim [30]), is that depressive patients had significantly higher plasma levels of IL-12 than did normal controls, and IL-12 levels were decreased after sertraline treatment. The finding of increased IL-12 in patients with major depression, together with increased IL-2, may be considered as additional evidence for activation of Th1-type immune response during major depression.

In consistency with previous reports (Kubera et al. [25], Kubera et al. [20], Mikova et al. [31], Tuglu et al. [32]), our results suggested that in comparison with healthy controls, patients with major depression have significantly higher levels of TNF-*α*, which decrease after sertraline treatment. Likewise, Lanquillon et al. [18] reported that the production of TNF*α* by peripheral blood mononuclear cells is significantly higher in depressed patients than in normal controls. However, other authors (Brambilla and Maggioni [12], Haack et al. [33]) reported no alteration found in TNF-*α* level in patients with major depression. The variable results, to some extent, may be associated with the characteristics of the illness, as well as the gender of the patients.

In the current study, TGF-*β*1, the Th3 cytokine, showed lower value in patients with major depression than healthy controls, and plasma TGF-*β*1 levels were significantly increased after 8-week treatment with sertraline as previously reported (Lee and Kim [30], Myint et al. [28]). Since TGF-*β*1 has multiple suppressive actions on T cells, B cells, macrophages, and other cells, sertraline may change the proinflammatory/anti-inflammatory cytokine balance via increased TGF-*β*1 levels in major depression.

Similar to our results, high levels of MCP-1 have been reported previously in untreatedpatients with major depression (Rajagopalan et al. [34]). Conversely, some data are inconsistent (Motivala et al. [35]), but limitations of this study which must be addressed are due to the relatively small size of the groups.

Some potential limitations of the current study should be noted. First, the sample size is too small and the design is too simple. Due to our research capabilities and protocol, the samples from the controls could not be obtained at the postassessment. A further replication study that examines the effects of each antidepressant on these immunological parameters in larger numbers of patients will be warranted. Second, since we collected the plasma samples only in the beginning and at the end of an 8-week treatment period, some significant changes in cytokine and chemokine levels could have been missed. Third, it is still unclear whether altered cytokine and chemokine levels are responsible for the provocation of depression or merely represent secondary features of the illness. We also would like to declare that it is obvious that relying upon diagnosis by clinicians is less optimal than structured interview. This is one of the limitations of the study. Finally, our results show the effect of sertraline only in responders group.

In conclusion, our results indicate that Th1, Th2, and Th3 are altered in the depressed patients. Some of them may have been corrected by sertraline treatment. These results support the concept that depressive disorders have been associated with changes of various aspects of the immune response, both immunoactivation and immunosuppression.

## Figures and Tables

**Table 1:** Clinical and immunological features in patient and control groups.

Parameters	Patients with major depression (*n* = 23)	Healthy controls (*n* = 25)	*t*	*P*
Age, years	34.78±7.42*	34.32±7.80	0.210	NS
Hamilton depression rating scale	28.39±4.53	4.20±1.80	23.927	<.001
Interleukin-2 (pg/ml)	33.45±6.37	14.65±2.79	13.053	<.001
Interleukin-4 (pg/ml)	3.43±0.51	7.80±1.75	11.917	<.001
Interleukin-12 (pg/ml)	16.50±3.74	6.17±1.86	11.978	<.001
Tumor necrosis factor-*α* (pg/ml)	77.68±16.21	36.04±12.63	9.972	<.001
Transforming growth factor-*β*1 (ng/ml)	1.52±0.21	3.09±0.46	15.361	<0.001
Monocyte chemotactic protein-1 (pg/ml)	84.54±12.54	48.09±8.19	11.647	<0.001

*Mean ± SD.

**Table 2:** Effect of sertraline therapy on clinical and immunological features in patient with major depression.

Parameters	Before sertraline therapy	After sertraline therapy	*t*	*P*
Hamilton depression rating scale	28.39±4.53	13.57±2.21	28.885	<.001
Interleukin-2 (pg/ml)	33.45±6.37	19.29±4.05	10.113	<.001
Interleukin-4 (pg/ml)	3.43±0.51	6.25±1.17	14.414	<.001
Interleukin-12 (pg/ml)	16.50±3.74	10.45±3.61	13.821	<.001
Tumor necrosis factor-*α* (pg/ml)	77.68±16.21	53.85±8.30	7.199	<.001
Transforming growth factor-*β*1 (ng/ml)	1.52±0.21	2.68±0.62	10.310	<.001
Monocyte chemotactic protein-1 (pg/ml)	84.54±12.54	54.79±10.33	10.440	<.001
